# Artificial Intelligence-Based Prediction of Cardiovascular Diseases from Chest Radiography

**DOI:** 10.3390/jimaging9110236

**Published:** 2023-10-26

**Authors:** Juan M. Farina, Milagros Pereyra, Ahmed K. Mahmoud, Isabel G. Scalia, Mohammed Tiseer Abbas, Chieh-Ju Chao, Timothy Barry, Chadi Ayoub, Imon Banerjee, Reza Arsanjani

**Affiliations:** 1 Department of Cardiovascular Medicine, Mayo Clinic, Phoenix, AZ 85054, USApereyra.milagros@mayo.edu (M.P.); abbas.mohammedtiseer@mayo.edu (M.T.A.); barry.timothy@mayo.edu (T.B.);; 2 Department of Cardiovascular Medicine, Mayo Clinic, Rochester, MN 55905, USA; 3 Department of Radiology, Mayo Clinic, Phoenix, AZ 85054, USA; banerjee.imon@mayo.edu

**Keywords:** artificial intelligence, chest radiography, cardiovascular diseases, deep learning

## Abstract

Chest radiography (CXR) is the most frequently performed radiological test worldwide because of its wide availability, non-invasive nature, and low cost. The ability of CXR to diagnose cardiovascular diseases, give insight into cardiac function, and predict cardiovascular events is often underutilized, not clearly understood, and affected by inter- and intra-observer variability. Therefore, more sophisticated tests are generally needed to assess cardiovascular diseases. Considering the sustained increase in the incidence of cardiovascular diseases, it is critical to find accessible, fast, and reproducible tests to help diagnose these frequent conditions. The expanded focus on the application of artificial intelligence (AI) with respect to diagnostic cardiovascular imaging has also been applied to CXR, with several publications suggesting that AI models can be trained to detect cardiovascular conditions by identifying features in the CXR. Multiple models have been developed to predict mortality, cardiovascular morphology and function, coronary artery disease, valvular heart diseases, aortic diseases, arrhythmias, pulmonary hypertension, and heart failure. The available evidence demonstrates that the use of AI-based tools applied to CXR for the diagnosis of cardiovascular conditions and prognostication has the potential to transform clinical care. AI-analyzed CXRs could be utilized in the future as a complimentary, easy-to-apply technology to improve diagnosis and risk stratification for cardiovascular diseases. Such advances will likely help better target more advanced investigations, which may reduce the burden of testing in some cases, as well as better identify higher-risk patients who would benefit from earlier, dedicated, and comprehensive cardiovascular evaluation.

## 1. Introduction

Chest radiography (CXR) is the most frequently performed radiological test worldwide due to its wide availability, non-invasive nature, low cost, and capacity to diagnose numerous lung and mediastinal diseases [1]. The cardiovascular structures are an important component of this test, as cardiac chambers and vessels are displayed by them. Most CXRs are reported as normal because they are performed to rule out specific diagnoses, such as lung processes. However, sometimes CXRs considered normal may show minor or subclinical abnormalities that could provide critical information for diagnosis and prognosis [2].

The ability of CXR to diagnose cardiovascular diseases, give insight into cardiac function, and predict cardiovascular events is often underutilized and not clearly understood [1]. Recent investigations have challenged some long-held medical views regarding traditional applications of CXR to diagnose cardiovascular conditions. It has been demonstrated that this test has only moderate accuracy in the diagnosis of congestive heart failure (HF) in patients presenting to the emergency department with acute dyspnea [3]. Additionally, the cardiothoracic ratio, a traditional marker of cardiac chamber dilation, may not be precise for the estimation of the left ventricle systolic function in patients with HF [4]. Another pitfall is inter- and intra-observer variability in CXR reading, which is inherent in the clinician assessment of imaging-based tests [5]. The assessment of specific chamber abnormalities on the cardiac silhouette on CXR, such as right ventricular, left atrial, or pulmonary artery size, is also often challenging for the general clinician. Finally, CXR provides a 2D representation of the cardiac silhouette and is not able to provide specific information about cardiac function.

Considering these limitations, more sophisticated tests are generally needed to assess cardiovascular function and diagnose cardiac conditions. These investigations, including echocardiography and cardiac magnetic resonance (CMR), are not only significantly more expensive than CXR but also require specialized operators, limiting their availability to some regions of the world [6]. Considering the sustained increase in the incidence of cardiovascular diseases, it is critical to find an accessible, fast, and reproducible test to help diagnose these frequent conditions.

**Figure 1:**
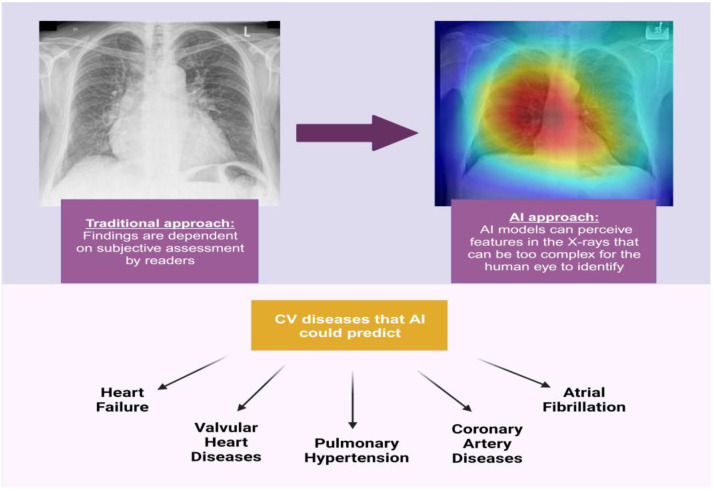
Recent investigations have challenged some long-held medical views regarding traditional applications of CXR to diagnose cardiovascular conditions. Novel AI models rely on automated feature extraction from images and do not depend on human decision-making skills.

Recently, there has been an expanded focus on the application of artificial intelligence (AI) with respect to diagnostic cardiovascular imaging [7]. AI models can be trained to identify features in imaging studies that are too subtle for the human eye to detect and to investigate relationships between those imaging patterns and clinical metadata [8]. These AI models are frequently based on convolutional neural networks (CNNs) for image analysis models and are particularly powerful, as they automate feature extraction from images and do not rely on human decision-making skills (Figure 1) [9]. By correlating clinician information with data from AI algorithms, the precision of imaging methods can be improved and inter- and intra-operator variability minimized [7]. These automated models including imaging patterns can be used to predict clinical information, diseases, and outcomes in a more reliable, easier, and faster way than the traditional clinician-based interpretation approach. Such models can be especially useful in regions that lack the availability of specialists or improve workflow accuracy in high-throughput practices.

Wide availability, low cost, low radiation dose, and simple image acquisition make CXR an important test for the application of AI technologies for the detection of cardiovascular diseases and for prognostication. Indeed, features from CXR have been analyzed in novel AI-based studies to predict clinical information, including mortality, cardiovascular morphology and function, coronary artery disease (CAD), valvular heart diseases (VHDs), aortic diseases, arrhythmias, pulmonary hypertension (PH), and HF [1]. This narrative review aims to summarize and analyze the available data regarding the use of CXR in AI models for the prediction of cardiovascular conditions and outcomes.

## 2. Results

### 2.1. Heart Failure

HF is rapidly increasing globally and is associated with high rates of morbidity and mortality. It imposes a heavy economic burden on healthcare costs [10]. This clinical syndrome is due to the inability of the heart to pump blood to meet systemic demands, or takes place when the heart is able to meet systemic demands but does so at the expense of elevated filling pressures [11]. Therefore, assessment for cardiac filling pressures is pivotal during the diagnostic workup for HF. The gold standard procedure to detect increased left ventricular filling pressure is the measurement of pulmonary arterial wedge pressure (PAWP) by right heart catheterization (RHC). However, due to its invasive nature, this diagnostic test cannot be performed routinely. Transthoracic echocardiography (TTE) can assess diastolic function and estimate filling pressures using several parameters; however, it is not diagnostic as a standalone test and must be taken in the context of the whole clinical picture. TTE is also subject to significant inter-observer variation, and assessment of diastolic function can be challenging and requires expertise. Therefore, HF diagnosis is currently based on a combination of several techniques, including medical history, physical examination, TTE, and laboratory markers [12].

Despite the unquestionable clinical value of CXR, the sensitivity and specificity of this method for diagnosing and grading HF are relatively low, thus necessitating the development of applications of AI in this field [13]. In 2020, Matsumoto et al. published one of the earliest experiences of the use of deep learning (DL) algorithms for diagnosing HF by using CXR images. The authors included 952 CXRs from a labeled database, and the images were verified and relabeled by two expert cardiologists [14]. After using data augmentation and transfer learning, the model reached an accuracy of 82% in identifying patients with HF, thus supporting the application of AI technologies to CXR in this scenario.

Historically, several attempts have been made to estimate PAWP by using non-invasive tests, with suboptimal or inconclusive results [15]. Regarding AI models, Hirata et al. conducted a study to detect elevated PAWP by applying DL techniques to CXR by including 1013 consecutive patients with both a RHC and a CXR [16]. The authors trained five CNN models to identify patients with elevated PAWP (>18 mmHg) based on the automated analysis of CXRs. The sensitivity and specificity of the AI model were not significantly different from those of the brain natriuretic peptide (BNP) and left ventricular diastolic dysfunction grade by TTE (AI model: 0.77 vs. BNP: 0.77 vs. TTE model: 0.70; *p* value not significant for all comparisons). The AUC of the automated CXR model was also significantly more accurate than the AUC of the traditional CXR cardiothoracic ratio. The model using three conventional parameters (BNP, diastolic dysfunction by TTE, and cardiothoracic ratio) was significantly enhanced by the addition of the AI prediction (AUC from 0.80 to 0.86; *p* = 0.041). Therefore, the AI-based model using CXR was shown to be a useful method to estimate high PAWP with comparable accuracy to traditional laboratory and TTE parameters that could potentially reduce intra- or inter-observer variations and error.

Another crucial aspect of HF diagnosis is the assessment of left ventricular systolic dysfunction, as this evaluation provides important information related to the prognosis and management of this condition [17]. It is not possible to perform a direct measurement of left ventricular ejection fraction (LVEF) from CXR because of the inability of this method to evaluate systolic and diastolic chamber dimensions and view function [9]. However, several features displayed on CXR could be associated with left ventricular dysfunction. Considering this, Hsiang et al. aimed to develop a DL model to identify left ventricular systolic dysfunction (LVEF < 35%) by using an automated CXR analysis [18]. The authors included a dataset of 90,547 CXRs with corresponding LVEFs from TTE and demonstrated AUCs of 0.88 and 0.87 in internal and external validation cohorts, respectively. Additionally, in patients incorrectly classified as having LVEF < 35%, the authors found a significant increase in the risk of developing a cardiomyopathy (HR = 3.91), and patients correctly classified by their model as having systolic dysfunction had an increased risk of mortality from all causes (HR = 1.40) and mortality from cardiovascular causes (HR = 3.02). Therefore, these findings could potentially help achieve an earlier diagnosis of left ventricular systolic dysfunction by using a widely available, simple, and inexpensive screening tool.

### 2.2. Pulmonary Hypertension

A prompt and precise diagnosis of PH is pivotal to ensuring that these patients receive timely treatment for this severe condition [19]. Currently, RHC is still the gold standard for the definitive diagnosis and hemodynamic assessment of patients with PH. RHC is paramount both in differentiating pulmonary arterial hypertension from patients with PH from left heart disease and in defining the severity of the disease. TTE is typically utilized as the first tool for its screening. Even when widely available in high-income countries, TTE assessment of PH can be relatively expensive and requires operators with specific knowledge and expertise of the condition [20]. Accessibility to recommended diagnostic modalities for PH is limited, especially in remote facilities with limited access. A simple, low-cost, and globally available tool for non-invasive PH screening would be of paramount importance.

Although certain findings can be suggestive of possible PH in patients undergoing CXR when suspected of the disease, known features of CXR have low sensitivity and specificity [21]. Considering this, Kusunose et al. aimed to evaluate the application of AI to CXR to identify patients with elevated pulmonary artery pressure (PAP) and predict their prognosis [22]. The authors included 900 consecutive patients with suspected PH by TTE and CXR and trained a CNN to detect patients with a mean PAP > 20 mmHg and to predict their risk of admission or occurrence of HF. The AI model was compared to CXR measurements made by physicians who were blinded to RHC data (widening of the hilum, projection of the right heart border (PRHB), ratio of hilar widening to chest widening, addition of PRHB to the hilum) and human (“eyeball”) visual assessment performed by a consensus of 10 physicians who were also blinded to RHC results. The AUC of the AI model for the detection of high PAP was significantly more accurate than the AUC of traditional CXR measurements and visual assessment (0.71 vs. 0.60 and 0.63, *p* < 0.05 for all comparisons). Patients in whom PH was correctly predicted by the AI model also had twice the higher risk of HF than patients without PH by DL model prediction. Although the study showed limited performance when applying AI to CXR to identify elevated PAP, it laid the groundwork for future and larger studies to further develop an AI model that would be inexpensive and universally accessible to opportunistically screen for PH, particularly in under-resourced areas.

The same group led by Kusunose et al. used the previously developed CXR-AI algorithm to investigate whether this model could predict exercise-induced PH in scleroderma patients or patients with mixed connective tissue diseases [22]. The authors recruited 142 patients who underwent a 6 min walk stress echocardiography test and defined exercise-induced PH as an abnormal cardiac output response with an increase in mean PAP. Multivariate regression demonstrated that gender, mean PAP at rest, and the probability of PH based on the AI algorithm were independent predictors of exercise-induced PH. Adding baseline clinical variables (age, gender, and PAP at rest) enhanced the prediction of the AI model (AUC: from 0.65 to 0.74; *p* = 0.046). The authors concluded that the use of this DL model based on CXR could have the potential to identify patients with exercise-induced PH in this clinical setting.

### 2.3. Coronary Artery Disease

CAD remains the leading cause of preventable death worldwide [23], highlighting the importance of screening and risk factor identification [24]. Currently, the risk for CAD is estimated by the complex interplay of several clinical, laboratory, and imaging parameters, and the risk of coronary events frequently persists despite appropriate control of known risk factors [25]. There is a need to develop and apply more accurate and reproducible methods to predict the risk of CAD and to better guide the performance of more invasive coronary diagnostic investigations.

D’Ancona et al. developed a DL model for the detection of significant CAD by analyzing retrospectively the CXR and coronary angiography of 7728 patients referred for angina to a single institution [26]. Only posteroanterior (standing) and anteroposterior (sitting) CXRs were included in the study, and severe CAD was defined with a binary classification (stenosis severity of ≥70% for non-left main vessels and ≥50% for left main coronary artery). In the binary logistic regression, the DL CXR model was the strongest predictor for severe CAD (*p* < 0.001; OR: 1.04) with an AUC of 0.73. Adding angina status to the DL CXR model enhanced its capability of predicting severe CAD (AUC 0.77). The authors concluded that this AI-based algorithm could be used for the pre-test prediction of significant CAD probability in patients with angina symptoms.

The coronary artery calcium (CAC) score is used in clinical practice to quantify the degree of atherosclerotic coronary artery calcification and has been shown to add prognostic value in estimating cardiovascular events in patients with intermediate risk for CAD. Kamel et al. studied the ability of CNN on CXR to predict CAC scores and cardiovascular risk using retrospective data from 1689 CXRs in patients who underwent cardiac CT and CXR within the same year [27]. Models were trained using binary classifications: (1) non-zero vs. zero CAC score, (2) presence or absence of calcium in each coronary artery, and (3) CAC scores above or below different thresholds. For the zero vs. non-zero CAC score classification, the AI model obtained an AUC of 0.73 (frontal CXR) and 0.70 (lateral CXR). For the binary classification of CAC scores above or below 100, the AUC was 0.74. Moreover, frontal CXRs that predicted a non-zero CAC score also had the ability to predict 10-year atherosclerotic cardiovascular disease, and multivariate regression analysis showed that the model could predict a non-zero CAC score independent of traditional cardiovascular risk factors. This modest accuracy of the AI model to predict CAC scores needs to be enhanced and evaluated by future studies in this field.

### 2.4. Valvular Heart Diseases

VHDs are a spectrum of pathologies in which one or more cardiac valves are damaged and become leaky (regurgitation) or tight (stenosis), preventing the correct functioning of the heart and leading to HF and other complications [28]. Rheumatic heart disease (RHD) remains the most common cause of VHD, primarily in low- and middle-income countries, because of untreated or undertreated streptococcal infections [29]. According to the Global Burden of Disease Study 2019, RHD affected 40.5 million people in 2019 and led to 306,000 global deaths [30]. In high-income nations, degenerative calcific aortic stenosis is the leading cause of VHD, with an ever-increasing burden due to an aging population along with an increased prevalence of atherosclerosis [29]. The burden of VHD is expected to keep rising in the upcoming years, which compels physicians and the healthcare industry to put the focus on creating novel, speedy, unchallenging, and widely available diagnostic tools to enable prompt treatment and improve morbidity and mortality. AI algorithms have been used in many diagnostic approaches for VHD that range from assisted cardiac auscultation to imaging tools such as CXR, TTE, and CMR [31]. Although cardiac auscultation remains a convenient, cost-effective, and rapid way to detect VHDs at the bedside, it still has low sensitivity and specificity, leading to the need for imaging studies [32].

In a study conducted by Kim et al., a DL-based automatic CXR cardiovascular border (CB) analysis algorithm was developed using CXRs of 816 normal and 798 VHDs (aortic stenosis, aortic regurgitation, mitral stenosis, and mitral regurgitation) for the diagnosis and quantification of VHDs. Comparisons of the baseline characteristics, TTE findings, and CB parameters measured by clinicians and by the AI model were performed. The CB parameters defined by the DL algorithm reached excellent reliability with an intra-class correlation coefficient >0.98, which was comparable to manual CB drawing. Moreover, all AI-based CB parameters were significantly greater in VHDs than in the normal controls and showed statistically significant correlations with various TTE measurements, including LVEF, left ventricular volumes, and left atrial dimensions [33].

In the last two years, the group led by Ueda et al. has published three different articles on AI algorithms for the detection of VHD from CXR. In one of these publications, the development of three DL models (InceptionV3, ResNet50, and DenseNet121) to detect aortic stenosis from CXR was analyzed. The authors retrospectively collected 10,433 CXRs from 5638 patients who had undergone TTE and labeled the CXRs as positive or negative for aortic stenosis. Soft voting was used to create an ensemble model, which summed the weighted means of the probability scores of the three models. Among all the models, the ensemble one showed the highest overall performance with an AUC, sensitivity, specificity, and accuracy of 0.83, 0.83, 0.69, and 0.71, respectively [34]. A visualization analysis showed that the models focused not only on the aortic valve but also on several left ventricular regions, potentially improving classification accuracy with a higher severity of aortic stenosis. Another retrospective study from this group used similar methods to develop an AI-based model to detect mitral regurgitation from CXR. The AI-based model had an AUC, sensitivity, specificity, and accuracy of 0.80, 0.71, 0.74, and 0.73, respectively, with a sensitivity for determining degenerative mitral regurgitation of 84% and functional mitral regurgitation of 89% in the test dataset [35]. In the study, the visualization analysis showed that the model focused more on the left atrium and the hilum.

Recently, this same group published a novel multilabel DL model to classify several VHDs, including mitral regurgitation, aortic stenosis, aortic regurgitation, mitral stenosis, tricuspid regurgitation, and pulmonary regurgitation from CXR. The authors included a cohort of 16,946 patients with a total of 22,551 CXRs, which were labeled using TTE reports. The AUCs for the classifiers of VHD at the none–mild vs. moderate–severe cutoff were 0.89 for mitral regurgitation, 0.83 for aortic stenosis, 0.83 for aortic regurgitation, 0.86 for mitral stenosis, 0.92 for tricuspid regurgitation, and 0.86 for pulmonary regurgitation [1].

Overall, these results indicate that CXRs may have intrinsic features (both detectable to the eye and subclinical) that can help with the diagnosis of VHDs. Automated image interpretation can enable more accessible and accurate cardiovascular assessments and alleviate disparities in the cardiovascular care of patients with these common conditions, as well as allow for earlier detection and, therefore, better management [34,35,36].

### 2.5. Aortic Diseases

Aortic diseases, especially aortic dissection (AD), which is a disastrous clinical event, are intimately associated with conditions leading to increased aortic wall stress and aortic media abnormalities [37,38]. Numerous studies have suggested an incidence of AD of 2.6 to 3.5 cases per 100,000 person-years, which translates to 6000 to 10,000 cases annually in the US [39,40]. A higher AD incidence has been reported in other cohorts [41,42]. AD is oftentimes misdiagnosed in the emergency department, posing catastrophic consequences for patients as mortality approaches 50% in the first 48 h after onset [43,44,45,46].

A study conducted by Liu et al. aimed to develop DL models based on CXR and ECG features to detect AD and then compare the performance of trained AI models with physicians’ impressions. The development cohort included 35,270 patients who were divided into five subgroups for five-fold cross-validation. In total, 43,365 CXRs were included in the development set, of which 74 were type A AD and 49 were type B AD [47]. The AI algorithm based on ECG and CXR reached an AUC of 0.96 for detecting AD type A, with a sensitivity of 100.0% and a specificity of 81.7%. The DL model based on CXR alone had a sensitivity of 94.4% in the identification of type A AD; however, the sensitivity for type B AD was only 50.0%. This last finding could be potentially explained by the larger areas affected by type A AD and by the fact that there could be a lack of involvement of the thoracic aorta in type B cases. Moreover, an additional analysis demonstrated that the application of the integrated AI algorithms to patients with chest pain and a D-dimer analysis further improved the accuracy of AD diagnosis.

A recent publication from Lee et al. assessed the diagnostic accuracy of DL techniques for the detection of thoracic AD based on CXR. A total of 3331 images with 716 AD cases were included. The results demonstrated that the diagnostic accuracy of the AI model was 90.2%. In the study, precision (which denoted the fraction of correct positive detection of thoracic AD) was estimated to be 75.0%. The authors also reported a recall of 94.4%, which depended on the percentage of relevant cases accurately classified by the algorithm, and an F1-score of 83.6%, which denoted the harmonic mean of precision and recall [48].

Overall, these studies established the basis for a future DL model that could predict specific diseases like AD, which is a challenging clinical entity for all-level physicians.

### 2.6. Atrial Fibrillation

Although CXRs provide useful anatomical information for a wide spectrum of cardiovascular conditions, arrhythmias remain one of the most difficult diseases for radiologists to predict from these imaging tests. Considering the common prevalence and clinical significance of atrial fibrillation with associated risk for stroke and potentially HF, the application of DL models to CXR has also been investigated for the detection of this arrhythmia. Atrial fibrillation is clinically associated with atrial enlargement, which may be visually appreciated on CXR only when the atrial dilatation is generally severe.

Matsumoto et al. retrospectively analyzed CXRs from patients who also underwent TTE and ECG at a single institution [49]. In total, 13,868 CXRs from 7047 patients were assessed (11,105 images for the training dataset, 1388 images for the validation dataset, and 1375 images for the test dataset). Only posteroanterior CXRs obtained in the standing position within 30 days of the TTE were analyzed. If more than one eligible CXR was available, all the CXRs were utilized. The performance of the validation dataset showed an AUC of 0.81, with a sensitivity of 0.76 and a specificity of 0.75. For the test dataset, the AUC was 0.80, the sensitivity was 0.70, and the specificity was 0.74. Notably, for the true-positive cases, a visual evaluation was performed, indicating that the area that the AI model focused on was the left atrium, followed by the right atrial region. However, it is still not completely certain how the visual interpretation of the silent maps correlates with the AI model. Future technical developments in AI are needed to better understand the basis of the model’s decisions. The authors concluded that this AI model could robustly predict atrial fibrillation from CXRs, providing physicians with additional ways to identify patients with this frequent condition.

### 2.7. Clinical and Cardiovascular Outcomes

There has been work to study the ability of DL algorithms to predict long-term prognosis from CXR images. Lu et al. developed a CNN model to predict 12-year mortality from CXRs using more than 50,000 images from two multicenter clinical trials: the Prostate, Lung, Colorectal, and Ovarian Cancer Screening Trial (PLCO) for development and testing and the screening radiography arm of the National Lung Screening Trial (NLST) for external validation [2]. The mortality risk score from the DL CXR model was established as very low, low, moderate, high, and very high, according to CNN predictions. They showed that images in the very-high-risk group had an increased risk of mortality compared to those in the very-low-risk group, confirming a consistent association between the AI-based risk score and mortality in both the included clinical trials (HR 18.3 (95%CI, 14.5–23.2) for PLCO and 15.2 (95%CI, 9.2–25.3) for NLST; both *p* < 0.001). This association was also significant after adjusting for radiologists’ findings and risk factors (age, sex, and diabetes), and notably, the association was also significant when analyzing non-cancer cardiovascular death alone. These findings suggest that the DL CXR score could differentiate patients at low and high risk for cardiovascular death by a single CXR image, therefore identifying patients in whom a more comprehensive cardiac evaluation would be warranted and allowing for the initiation of early preventative measures, including lifestyle changes.

Ieki et al. utilized AI to predict age and cardiovascular disease from CXRs. They trained a deep neural network model using more than 100,000 CXRs to predict the age of the patients and then applied their model to patients admitted for HF and patients hospitalized in an intensive care unit for cardiovascular diseases [50]. The AI-based prediction using CXRs precisely estimated the real chronological age of the patients, with higher accuracy than the estimates performed by expert physicians and radiologists. Moreover, a higher “CXR age” was associated with worse clinical outcomes for HF hospitalized patients (HF readmissions and all-cause mortality) and was associated with a worse prognosis in those cases admitted to the intensive care unit with cardiovascular disease. The authors concluded that their tool could act as a robust indicator of patients’ age and that cardiovascular outcomes may be predicted by using a simple CXR in patients with cardiovascular conditions.

## 3. Discussion

In summary, the studies reviewed in this article for the use of AI-based tools applied to CXR for the diagnosis of cardiovascular conditions and prognostication show early progress and have the potential to transform clinical care. AI-analyzed CXRs could be utilized in the future as a complimentary, easy-to-apply technology to improve diagnosis and risk stratification for cardiovascular diseases. Such advances will likely help better target more advanced and expensive investigations, which may reduce the burden of testing in some cases, as well as better identify higher-risk patients who would benefit from earlier, dedicated, and comprehensive cardiovascular evaluation.

CXR remains a key diagnostic test for the assessment of chest structures, including airways, lungs, mediastinum, heart, pleura, and chest wall. It is one of the most widely used diagnostic imaging techniques because it is fast to perform, relatively inexpensive, and non-invasive and has low radiation exposure. CXR provides important anatomical information that helps guide clinical management. It also contains data pertaining to body habitus, pulmonary condition, bone mineral density, and dimensions and calcification of cardiovascular structures. However, the characteristics or geometrical measurements derived from CXRs are often not highly sensitive or specific for the accurate prediction of cardiovascular diseases. CXRs do not currently provide sufficient information to permit standalone cardiovascular diagnoses in the absence of additional testing or risk stratification for clinical events. Therefore, its role in the diagnosis of cardiac diseases has become marginal, and other more sophisticated and expensive techniques have become more relevant.

**Figure 2:**
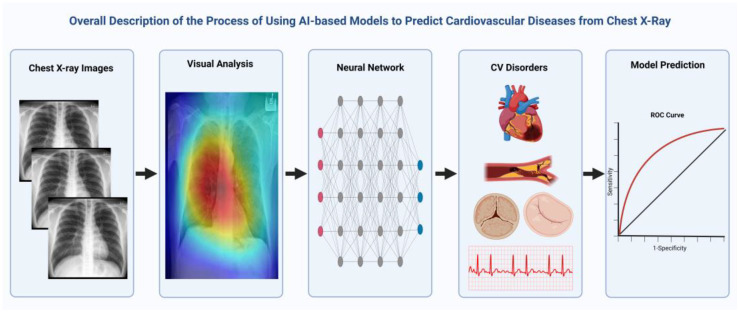
An example depicting the several neural networks that have been developed for detection of different cardiovascular conditions based on CXR analysis.

The introduction of AI-based analyses of medical images has already demonstrated the potential to detect features of several diseases that could remain overlooked by the human eye. DL technologies can indeed enhance imaging interpretation across a wide spectrum of diseases in the clinical setting by recognizing previously hidden characteristics or by identifying complex relationships that were previously unknown. As a result, predictions and associations that would not be otherwise supported by traditional methods can be made, allowing for readily available imaging analysis and opening possibilities for the accurate screening of a large portion of the population (Figure 2).

Although the capabilities of DL methods for automated feature extraction on CXR appear strong and promising, several limitations of these investigations need to be highlighted. As outlined, DL models “learn” features during the training process that may not be obvious to human observers, thus raising the concern of interpretability. The “black box” nature of AI models, meaning their capability to produce results without the ability to reverse-engineer, makes it difficult to understand the reasons why an AI algorithm is making certain predictions. Although visualization analysis can partially help explain the AI models’ decisions, it is still not possible to completely understand how the predictions are generated. This limitation could certainly affect the potential of AI algorithms to become widely applied in the clinical setting, and future research on the decision-making process of the DL models could provide additional knowledge and insight in this regard. As a future objective, AI models should be interpretable by developing a set of technologies and tools that enable human users to understand how AI models reach their outcomes. This should ensure the transition to fully understandable AI algorithms for users, who will be able to comprehend the features that the AI models extract from cardiac imaging. Among other advantages, this will allow users to understand if the image features extracted are more indicative of the consequences of the diseases or subtle early abnormalities that are present before the development of the diseases.

Moreover, most of the reviewed AI models were developed from single-center analyses or from trials, including a limited number of academic facilities from high-income countries. Therefore, external validation in different demographics and socioeconomic populations is still needed prior to generalizing any of these AI-based models in clinical practice. Furthermore, most of these AI algorithms are developed retrospectively and therefore need to be analyzed in prospective studies. These AI models are usually trained on labeled data that are derived from human-based interpretations, so the potential risk of error during labeling cannot be excluded; of course, if the models are trained on biased data, this could lead to biased conclusions.

## 4. Conclusions

The application of AI technologies to CXR images for the detection of cardiovascular diseases and the prediction of cardiac outcomes is promising. Predictions based on a simple, non-invasive, widely available, and inexpensive tool may help improve the accuracy of the diagnostic process and provide novel cardiovascular clinical risk prediction and prognostication. However, current limitations need to be addressed by future studies to ensure the accurate clinical implementation of such models.

## Data Availability

No new data were created as a consequence of this investigation. Data sharing is not applicable to this article.
